# From Xiaoke to diabetes mellitus: a review of the research progress in traditional Chinese medicine for diabetes mellitus treatment

**DOI:** 10.1186/s13020-023-00783-z

**Published:** 2023-06-22

**Authors:** Xianglong Meng, Xiaoqin Liu, Jiaying Tan, Qi Sheng, Dingbang Zhang, Bin Li, Jia Zhang, Fayun Zhang, Hongzhou Chen, Tao Cui, Minghao Li, Shuosheng Zhang

**Affiliations:** 1 Shanxi University of Chinese Medicine, Jinzhong, 030619 Shanxi China; 2 Shanxi Key Laboratory of Tradition Herbal Medicines Processing, Jinzhong, 030619 Shanxi China; 3 The First Affiliated Hospital of Hunan University of Chinese Medicine, Changsha, 410021 Hunan China; 4 Guangxi University of Chinese Medicine, Nanning, 530001 Guangxi China

**Keywords:** Xiaoke, Diabetes mellitus, Traditional Chinese medicine, Etiology and pathogenesis, Treatment guidelines

## Abstract

Diabetes mellitus (DM) is a chronic metabolic disorder characterized by hyperglycemia resulting from insulin secretion defects or insulin resistance. The global incidence of DM has been gradually increasing due to improvements in living standards and changes in dietary habits, making it a major non-communicable disease that poses a significant threat to human health and life. The pathogenesis of DM remains incompletely understood till now, and current pharmacotherapeutic interventions are largely inadequate, resulting in relapses and severe adverse reactions. Although DM is not explicitly mentioned in traditional Chinese medicine (TCM) theory and clinical practice, it is often classified as “Xiaoke” due to similarities in etiology, pathogenesis, and symptoms. With its overall regulation, multiple targets, and personalized medication approach, TCM treatment can effectively alleviate the clinical manifestations of DM and prevent or treat its complications. Furthermore, TCM exhibits desirable therapeutic effects with minimal side effects and a favorable safety profile. This paper provides a comprehensive comparison and contrast of Xiaoke and DM by examining the involvement of TCM in their etiology, pathogenesis, treatment guidelines, and other relevant aspects based on classical literature and research reports. The current TCM experimental research on the treatment of DM by lowering blood glucose levels also be generalized. This innovative focus not only illuminates the role of TCM in DM treatment, but also underscores the potential of TCM in DM management.

## Background

According to the 10th edition of the Global Diabetes Map, 537 million adults were diagnosed with diabetes mellitus (DM) worldwide in 2021, and this number is expected to rise to 643 and 783 million in 2030 and 2045, respectively [1]. A recent study on the prevalence and treatment of DM in China between 2013 and 2018 revealed that the prevalence in Chinese adults had increased from 10.95 to 12.4%. This trend is thought to be dues to an overall rise in DM risk factors, such as unhealthy diets, lack of physical activity, smoking, alcohol consumption, and obesity and overweight [2, 3]. Studies have demonstrated that Chinese people have weaker β-cell functionality and a high prevalence of obesity that is concomitant with insulin resistance (IR), contributing to the weak β-cell function which leads to an increase in the prevalence of DM [4]. The classification of DM includes type 1 (T1DM), type 2 (T2DM), or gestational (GDM), as well as distinct forms such as type 3 (T3DM) and type 3c (T3cDM) DM. T2DM accounts for 90% of all DM cases in the Chinese population. According to the Guidelines for the Prevention and Treatment of Type 2 Diabetes Mellitus in China (2020 Edition), the awareness (36.5%), treatment (32.2%), and control rates (49.2%) of DM in China have improved; however, those percentages remain relatively low [5].

The treatment strategy for DM is not only limited to reducing blood glucose, but also includes the control of blood pressure, blood lipids, and body weight; antiplatelet therapy; and lifestyle improvement. Treatment should follow the principles of individualized medicine; however, the current hypoglycemic drugs are both unsatisfactory in their therapeutic effects and not well-tolerated, and they cannot meet the needs of personalized treatment. The merits of TCM include treatment based on syndrome differentiation and a holistic view. Results from drug therapy, acupuncture, moxibustion, diet control, and other affiliated therapies, sufficiently demonstrate the efficacy of TCM in the overall regulation and treatment of multiple targets and in providing personalized medication. TCM can significantly ameliorate the clinical symptoms of DM and effectively prevent and treat its complications, which has been a common strategy for managing chronic metabolic disorders typified by DM.

No relevant description of DM can be found in TCM theory and clinical practice. Therefore, DM is often classified as “Xiaoke”. However, despite similarities between Xiaoke and DM regarding etiology, pathogenesis, and symptoms, the two diseases are not identical. Previous studies have frequently addressed Xiaoke and DM, but a comprehensive comparison of their similarities and differences has been lacking. Drawing on classical literature and contemporary research on TCM's efficacy in treating these conditions, this paper presents theoretical considerations, methodological innovations, and clinical applications as the epistemological foundations of TCM treatment for Xiaoke and DM. This is not only illuminates the role of TCM in DM treatment, but also underscores the potential of TCM in DM management.

## DM stemming from Xiaoke based on TCM principles

According to TCM theory, DM falls under the category of Xiaoke. Xiaoke, the disease was first named in *The Yellow Emperor’s Inner Classic (Huang Di Nei Jing)* [6]. The character pattern and meaning of Xiaoke indicate that the disease is closely related to water metabolism. Patients suffering from this condition experience dry mouth, abnormal water distribution within their body, and pathological changes in their metabolism [7]. In the medical practices of past dynasties, TCM physicians deepened their understanding of Xiaoke and gradually developed a more comprehensive understanding of its etiology, pathogenesis, diagnosis, and treatment.

### Pre-Qin and Han dynasties (pre-A.D.220): academic origin of Xiaoke

In the Yin and Shang dynasties, oracle bone inscriptions described the “urinary disease”, which is probably the earliest record of “Xiaoke” [8]. The earliest medical book, Prescriptions for *Formulas* for *Fifty-two Diseases (Wu Shi Er Bing Fang)*, details the symptoms associated with Xiaoke. *Huang Di Nei Jing* not only classified and named this disease but also provided additional records on its clinical symptoms, etiology, treatment, prognosis, and taboos practices, which indicates a theoretical foundation for preventing Xiaoke and the emergence of dietary therapy in later generations. *Jin Gui Yao Lve* represents the pioneering application of syndrome differentiation in TCM treatment for Xiaoke, which has continued to inform modern therapeutic strategies.

### Wei, Jin, Sui, and Tang dynasties (A.D.220 to A.D.907): academic development of Xiaoke

*The Ancient and Modern Records of Proven Formulas (Gu Jin Lu Yan Fang)* was the first to record patients with Xiaoke who had sweet urine, which was a breakthrough in the diagnosis of Xiaoke [9]. *Arcane Essentials from the Imperial Library (Wai Tai Mi Yao)* quoted from *Gu Jin Lu Yan Fang* and elaborated on the characteristics of Xiaoke, and divided it into three categories: Xiaoke, Xiaozhong and Xiaoshen [10].

### The five dynasties and Liao and Song dynasties (A.D.907 to A.D.1115): academic accumulation period of Xiaoke

Official medical books of the Song dynasty such as *Formulas from Benevolent Sages Compiled during the Taiping Era (Tai Ping Sheng Hui Fang)* and *Comprehensive Recording of Divine Assistance (Sheng Ji Zong Lu)* contain a plethora of theories and prescriptions that have been used in the treatment of Xiaoke. *Tai Ping Sheng Hui Fang* first explicitly proposes the term "Sanxiao", which are respectively for Xiaoke, Xiaozhong, and Xiaoshen, including modern Shangxiao (focus on the lungs), Zhongxiao (focus on the stomach), and Xiaxiao (focus on the kidneys) [11–13].

### Jin and Yuan dynasties (A.D.1115 to A.D.1368): academic contention period of Xiaoke

During the Sui and Tang dynasties, the kidneys were deemed the main target for the treatment of Xiaoke. In contrast, in the Song, Jin, and Yuan dynasties, it was proposed that the disease should be located according to the Sanxiao term. The most prominent medical teachers in the Jin and Yuan dynasties were Liu Wansu, Zhang Congzheng, Li Dongyuan, and Zhu Danxi, who presented unique perspectives on the comprehension and treatment of Xiaoke. [14–17].

### Ming and Qing dynasties (A.D.1368 to A.D.1912): blooming academic period of Xiaoke

New classification methods have emerged, for example, Zhang Jingyue classified Xiaoke as Yinxiao and Yangxiao while Qin Jingming broadly classified Xiaoke due to exogenous affection and internal damage [18]. Furthermore, the efficacy of Xiaoke treatment has been significantly enhanced by establishing a syndrome differentiation-based therapeutic system that advocates for warming and nourishing kidney Yang, as well as increasing the application of Qi and blood theory in Xiaoke treatment. In terms of nursing care for Xiaoke, Fang Yu in the Ming dynasty especially pointed out that patients with Xiaoke should “drink more water freely” [19].

### The Republic of China (A.D.1912 to A.D.1949): academic collision period between Xiaoke and DM

The concept of diabetes mellitus (DM) in Western medicine (WM) was first introduced to China during the Republic of China era. In TCM, it is believed that DM corresponds to Xiaoke symptoms, which may have originated from the *Records of Chinese Medicine with Reference to Western Medicine (Yi Xue Zhong Zhong Can Xi Lu)*, written by Zhang Xichun [20]. Zhang created a novel prescription named the Zicui decoction, which transformed the pancreatic lesions recognized at that time into “involving the spleen, thereby affecting the lung and kidney.” During the Republic of China period, the treatment method continued to be based on Xiaoke and followed traditional Xiaoke treatment experiences [21].

### Modern times (A.D.1949 to present): the initial academic period considering Xiaoke and DM

Professor Tong Xiaolin's "type-stage-syndrome differentiation" method, which combines disease with syndromes, is widely used in DM or Xiaoke treatment [22]. Yue Rensong et al. proposed a classification of DM and its complications into three stages: early, middle, and late. Zhou Kai et al. divided the occurrence and development of DM into four stages: primitive, prodromal, diabetic, and retrogressive. In addition, the authors also put forward the theory of combining physiques with stages [23].

**Fig. 1:**
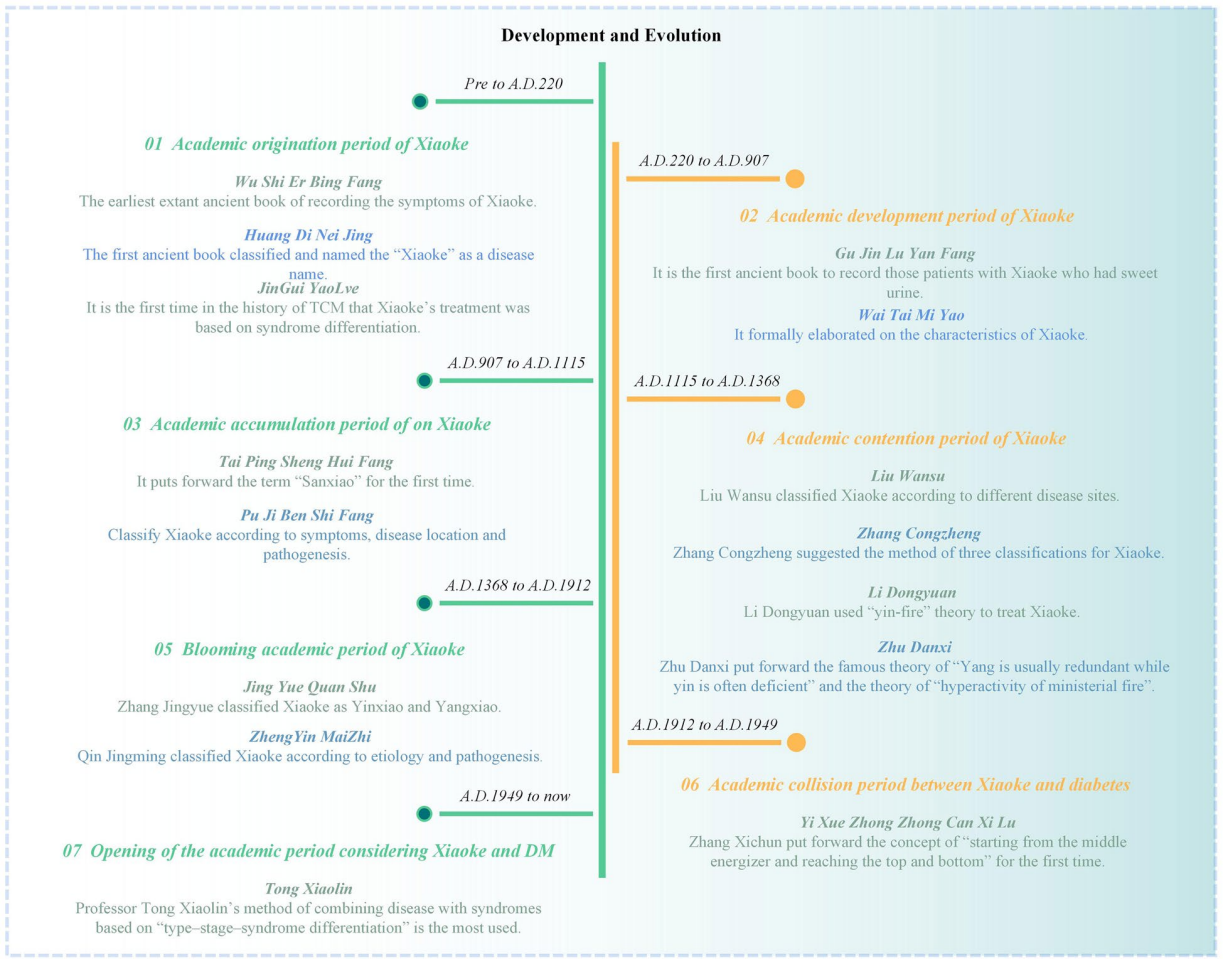
DM stemming from Xiaoke based on TCM principles

In TCM, Xiaoke is considered the initial etiology of DM, and the systematic understanding of DM in TCM starts from Xiaoke (summarized in Fig. 1). Modern TCM views Yin deficiency and dry heat as the primary pathogeneses of DM, with Yin deficiency being the cause and dry heat being the outer phenomenon. The specific details will be further elaborated in the following section.

## The role of TCM in the etiology and pathogenesis of DM

### Etiology of DM according to TCM

Understanding the etiology of DM began with a discussion of Xiaoke in *Huang Di Nei Jing*. Considering the research and summaries of physicians in past dynasties, the etiology of Xiaoke is divided into hereditary and acquired factors. Hereditary factors, namely genetic factors, include weakness of the organs and insufficient endowment; acquired factors are classified as exogenous affections and internal damage. The primary etiological factors of exogenous affection include wind, cold, heat, dampness, dryness and fire. Conversely, the primary causes of internal damage are uncontrolled diet and indulgence in sexual activities that lead to the depletion of kidney essence, as well as emotional disorders.

### Pathogenesis of DM according to TCM

#### Pathogenesis of the internal organs (Sanxiao theory)

Sanxiao is classified according to the location of diseased internal organs. Shangxiao indicates afflictions in the lungs and heart, Zhongxiao pertains to disorders in the spleen, stomach, liver, and gallbladder, while Xiaxiao denotes issues with the kidneys and bladder.

The Shangxiao pathogenesis is typically heart fire and lung dryness. Zhongxiao is characterized by spleen heat and liver Qi stagnation. Patients with Zhongxiao exhibit increased appetite and frequent hunger due to heat stagnation in the spleen and stomach. The primary pathogenesis of Xiaxiao is attributed to kidney deficiency. Therefore, compromised renal function in DM patients is not only a pathogenic factor but also a pathological outcome. Heat syndrome in the upper energizer causes thirst in patients resulting in hyperhydration, and the deficiency of Yin brings internal heat to the lower energizer, resulting in polyuria [24].

#### Pathogenesis theory of Qi, blood, and bodily fluids

Qi, blood, and bodily fluids are generated by internal organs and physiological processes. These substances serve as the foundation for human life activities. According to the theories of body fluid and Qi and blood, pathological changes in these substances play a crucial role in the development of Xiaoke.

The theory of bodily fluids describes “bodily fluids” as a collective term to represent all forms of water and fluids in the body, including the fluid within the internal organs, bodies, and orifices and their normal secretions. Xiaoke's pathogenesis can be attributed to insufficient bodily fluids or impaired transport and transformation thereof. Bodily fluid metabolism is closely linked with all internal organs; dysfunction in any one organ may result in abnormal metabolism and subsequent complications.

The theory of Qi and blood stipulate that Qi and blood are the essential elements that constitute the human body and maintain human life activities. They originate from the five organs and serve as a power source for all internal physiological functions. According to Xiaoke pathogenesis, Yin deficiency is fundamental while dryness-heat is incidental. Overconsumption of Qi can deplete Yin and negatively impact organ function. If left uncorrected for an extended period, this condition may result in a deficiency of Qi and blood, as well as impaired organ function, ultimately impacting the circulation of Qi and blood [25].

#### Modern theory: stage-type theory

The "stage-syndrome differentiation" method is commonly employed by scholars to categorize the stages and syndromes of DM. For example, Professor Tong Xiaolin divided DM into two categories, Pidan and Xiaodan. Obesity-related DM (obesity-DM) is in the Pidan category, which is common in patients with DM at this stage. While the typical development process of obesity-DM follows an “obesity or overweight-Pidan-Xiaoke/DM-Xiaoke complications” pathway, corresponding to most cases in T2DM. Emaciation DM falls under the Xiaodan category and encompasses T1DM, type 1.5 diabetes (LADA), and certain cases of T2DM in modern medicine. The pathogenesis underlying this form of DM is characterized by "Xiaodan-Xiaoke/DM-Xiaoke complications". Presently, the Guidelines for Clinical Evidence-Based Practice of TCM in Diabetes classify DM into three stages: pre-DM, DM, and DM complications [26].

#### Pre-DM stage

The pre-diabetes mellitus stage encompasses Qi, blood, phlegm, fire, dampness and food that contribute to the development of the disease. Impaired digestion is the foundation for Qi stagnation. Prolonged consumption of fatty, sweet and pungent foods or chronic spleen deficiency can lead to reduced digestive function in both the spleen and stomach resulting in abdominal distension. Stagnation in the spleen and stomach generates phlegm, which transforms into heat and eventually develops into Pidan. Emotional frustration rapidly induces stagnation of liver Qi, whose function is to regulate the flow of Qi. With stagnant liver Qi, blood circulation is impeded and body fluid circulation becomes poor, leading to internal organ stagnation and ultimately resulting in phlegm dampness. Phlegm dampness may lead to spleen dysfunction, resulting in Pidan. According to modern medical experts, complications during the pre-diabetes stage are typical of the Pidan category. Although early syndromes of DM vary, Yin deficiency and dry heat are considered the main manifestations [27].

#### DM stage

DM is characterized by heat, deficiency, Qi stagnation and food indigestion that is later converted to heat. It involves internal organs, primarily liver and stomach heat, as well as lung and intestinal heat. If the dryness-heat lingers for an extended period, it eventually leads to a deficiency of Qi, blood, Yin, and Yang [28].

In the TCM clinical practice, DM is classified as dryness-heat caused by deficiencies in Yin, both Qi and Yin, or both Yin and Yang, which corresponds to the early, middle, and late stages of DM, respectively. Among these classifications, the Qi and Yin deficiency corresponds to the main stage of DM. The pathogenesis of DM can be divided into deficiency and excess syndromes. The former manifests as Qi, Yin, and Yang deficiency while the latter primarily manifests as blood stasis and phlegm turbidity.

#### DM chronic complication stage

Types of common chronic DM complications are diabetic foot (DF), diabetic nephropathy (DN), diabetic peripheral neuropathy (DPN), and diabetic retinopathy (DR). Tong Xiaolin et al. suggested that most complications occur in the stage of “damage” [29]. At this stage, deficiency of Qi and Yin, water dampness, phlegm turbidity, and blood stasis are intertwined, and the flow of Qi and blood are turbulent; thus, pathogenic factors block the vessels, which is the key pathogenesis of early complications of DM [30]. The pathogenesis of the various complications differs. Deficiency in Qi and Yin is the underlying cause of DF, while dryness-heat, blood stasis, and phlegm turbidity are incidental factors [31]. DN results from a combination of deficiency in both Qi and Yin with blood stasis [32]. DPN can be attributed to fundamental deficiency along with incidental excess in blood stasis and phlegm turbidity [33]. DR is caused by a deficiency in Yin [34]. However, the integration of the organs and imbalances in Qi and blood functions can exacerbate the “damage”. Therefore, it is crucial to prevent and treat early complications of DM holistically by regulating the five organs to restore body functions [35].

**Fig. 2:**
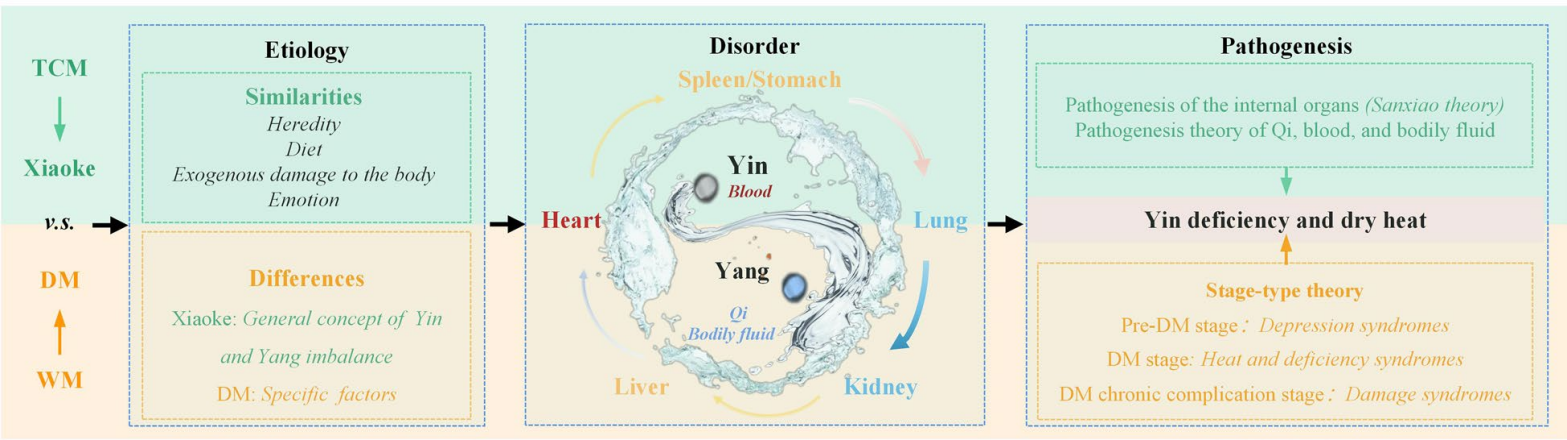
The role of TCM in the etiology and pathogenesis of DM

In summary, the systemic understanding of DM in TCM varies from local to general disease location and various stage-syndrome classifications. The disease location, course, etiology, and pathogenesis of DM are systematized and comprehensive. The systematic understanding of DM in TCM begins with Xiaoke. With extensive knowledge of pathogenic factors, such as phlegm dampness, blood stasis, and emotion, a holistic understanding of the pathogenesis and changes in Qi, blood, and bodily fluids has been achieved (summarized in Fig. 2). Treatment based on syndrome differentiation in Xiaoke has advanced from a discussion of the treatment of Sanxiao to the whole. Moreover, from the perspective of Qi, blood, bodily fluids, and emotions, starting from aspects of blood stasis, phlegm dampness, dampness-heat, and emotion, the treatment of DM is considered more comprehensively and a regimen based on syndrome differentiation has been established. In modern times, TCM incorporates WM diagnoses and pharmacological therapies, and the diagnosis of DM is based on the stage-syndrome classification. Although scholars have classified Xiaoke or DM according to the stage-type disease development process, its pathogenesis is summarized as Yin deficiency and dry heat.

## TCM treatment guidelines for DM

With the deepening understanding of the etiology and pathogenesis of DM in TCM, its treatment guidelines are gradually improved. The treatment guidelines are refined based on the pathogenesis described by Sanxiao theory, Qi, blood, and bodily fluids theory and stage-type theory.

### Treatment based on internal organs/Sanxiao

The primary pathogenesis of Xiaoke is attributed to Yin and body fluid deficiency as well as excessive dryness-heat. Therefore, its treatment principle involves clearing heat, moistening dryness, nourishing Yin and generating fluids. According to the distinct pathological characteristics of Shangxiao, Zhongxiao, and Xiaxiao, ancient physicians proposed the following treatment principles: When treating patients with Shangxiao, it is appropriate to moisten their lungs while simultaneously clearing the heat in their stomach. When treating patients with Zhongxiao, it is preferable to clear the heat in their stomach while also nourishing their kidneys. Finally, when treating patients with Xiaxiao, it is suitable to nourish their kidneys and tonify their lungs.

### Treatment based on Qi, blood, and bodily fluids

Xiaoke is regarded as a disorder of Qi function that disrupts the normal flow of Qi and blood, leading to excessive dryness-heat in the body. This results in fluid depletion, causing turbulent blood circulation and vessel blood stasis. The primary treatment principle focuses on replenishing Qi, promoting blood circulation, eliminating blood stasis, clearing heat, resolving phlegm, and strengthening spleen dampness [36, 37].

### Treatment based on stages

Pre-DM stage: Depression syndrome such as liver depression and Qi stagnation, and stagnation of spleen and stomach Qi may occur. The three major treatment strategies to restore a normal distribution of liver Qi and homeostatic function of ascending and descending spleen and stomach Qi are regulating liver Qi, strengthening spleen Qi, and lowering stomach Qi [38].

DM stage: DM primarily involves stagnated heat and deficiency syndromes. At this stage of heat, the treatment methods in addition to clearing away heat and purging fire, should be nourishing Qi and Yin [39]. In the deficiency stage, the treatment method is to clear heat, activate blood circulation, replenish Qi, and nourish the blood. The method that entails strengthening body resistance and correcting deficiency should be applied appropriately according to each case.

DM chronic complication stage: This DM stage is similar to the “damage” described by Xiaolin et al., which is characterized by several types of complications. In DF, the predominant syndrome is the deficiency of both Qi and Yin, which should be treated by replenishing Qi, nourishing Yin, activating blood circulation, and removing blood stasis [32]. For DN, the deficiency in Qi and Yin should be addressed by simultaneously stimulating both Qi and Yin [40]. The primary treatment for DPN involves promoting blood circulation and removing obstruction in the collaterals [39]. As for DR, treatment strategies include replenishing Qi, nourishing Yin, tonifying the liver and kidneys, promoting blood circulation, removing blood stasis, dispelling pathogenic wind, and brightening the eyes [41].

The initial stage of the disease is characterized by a combined depression in different aspects. The use of pungent and bitter herbs for dispersion and purgation, respectively, facilitates Qi flow and resolves phlegm. Prolonged depression can transform into heat. For patients with stagnant heat in the liver and stomach, it is more effective to alleviate depression and clear heat in the stomach. For those with excessive heat, bitter and sour drugs are recommended for glucose control. Dryness-heat can impair Yin and eventually lead to Qi, blood, Yin, and Yang deficiencies. Therefore, reinforcing Qi, nourishing blood, replenishing Yin, tonifying Yang, and moistening dryness are necessary.

The treatment guidelines for DM are gradually improving due to the continuous development and synergy between TCM and WM, which can vary depending on the patients during some stages of DM. Thus, treatment guidelines and strategies focus on alleviating deficiency and dryness-heat to nourish Yin and restore fluids regardless of the condition, DM or Xiaoke.

## Similarities and differences between Xiaoke and DM

There are similarities between Xiaoke and DM in the definitions, classifications, etiology, pathogenesis, diagnosis, and treatment, although not entirely consistent.

In terms of disease name definitions and classifications, Xiaoke is a TCM term that refers to a condition characterized by polydipsia, polyuria, polyphagia, weight loss, asthenia, and sweet urine, which is classified as Shangxiao, Zhongxiao, and Xiaxiao. DM is a WM disease name, referring to a chronic metabolic disorder characterized by hyperglycemia and caused by absolute or relative insufficient secretion of insulin and/or IR, which is classified into the pre-DM, DM, and DM chronic complication stages.

Although both conditions are related to factors such as heredity, diet, and exogenous damage to the body and emotion, the etiology of TCM is thought to involve all factors causing an imbalance in Yin and Yang. The etiology of Xiaoke includes hereditary factors such as insufficient endowment and weakness of the five zang-organs, acquired factors such as improper diet resulting in the accumulation of heat and damage to bodily fluids, disordered emotions causing the poor function of Qi, and excessive physical and sexual activities. The exogenous etiology of DM is more specific and includes risk factors such as an unhealthy diet, insufficient physical exercise, smoking, and alcohol consumption. IR is confounded by risk factors such as being overweight or obese.

Regarding the pathogenesis, dryness-heat caused by a deficiency in Yin is apparent in both diseases; however, DM is a complex metabolic condition. In the early stages of DM, there is typically no Yin deficiency, some patients with DM do not have common symptoms such as “three mores and one less” (polydipsia, polyphagia, polyuria, and weight loss).

**Fig. 3:**
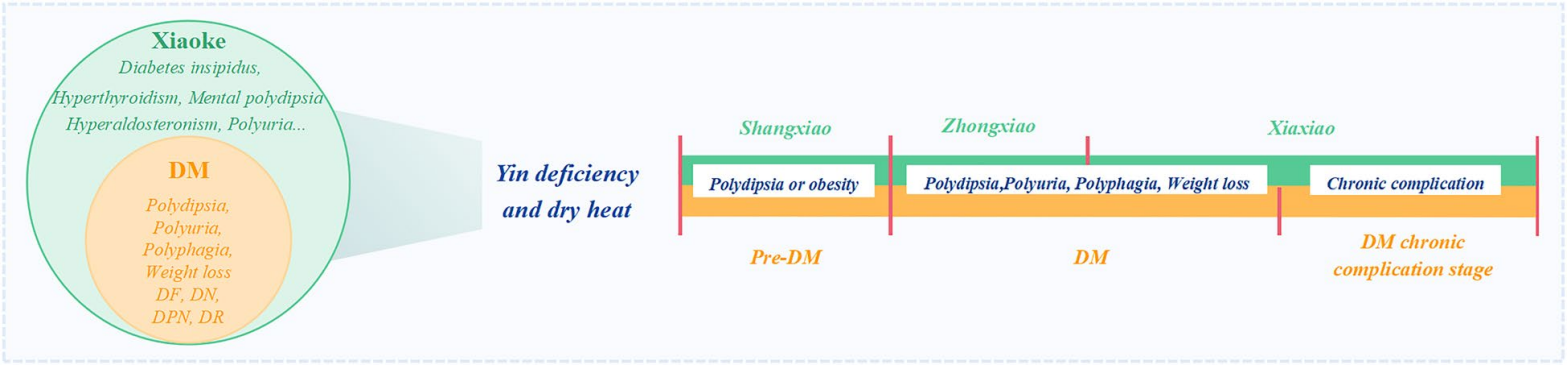
Similarities and differences between Xiaoke and DM based on TCM principles

In terms of diagnosis, Xiaoke is based on the presence of “Sanxiao” symptoms in patients. Early in the disease progression, typical Xiaoke manifestations include “three mores and one less” or sweet urine, similar to T1DM; during the pathological state, thirst and the desire to drink as well as frequent urination become the more prevalent symptoms, as in diabetes insipidus, hyperthyroidism, hyperaldosteronism, mental polydipsia, and polyuria, and other diseases in modern medicine [42]. Therefore, Xiaoke can only be diagnosed when patients with DM present with symptoms like “three mores and one less” [43]. The diagnostic criteria of DM include hyperglycemia and urinary glucose as the gold standard. The initial stage of DM consists of the excess syndrome and is characterized by “slow and stagnated Qi in the spleen and abnormal digestion and transportation,” which is related to Pidan. It progresses to Xiaoke when the “three mores and one less” symptoms appear, in association with “Xiaozhong,” kidney dryness, Gexiao, and “Xiaoke”. The “Xiaoke” stage is the progression from excess to deficiency or a mixture of poverty and excess, and the basic pathogenesis is dryness-heat due to a lack of Yin. During the DM complication stage, the organs are impaired and exhibit carbuncles, abscesses, and cataracts. These chronic complications of DM are remarkably similar to those of Xiaoke. Many patients do not have symptoms in the early stage, but their blood glucose is elevated when measured during physical examination. If the disease progresses to a Xiaoke state of “three mores and one less”, most patients would be diagnosed with middle or late stage-DM; thus, Xiaoke cannot be compared to modern DM. For DM, in the initial stage of TCM, the “syndrome differentiation of Sanxiao” is mainly used, and then the “symptom stage-type” is established [22]. Although this varies among individuals, the general treatment methods for Xiaoke involve removing heat, nourishing Yin, invigorating Qi, promoting bodily fluid production, benefiting Yin, supporting Yang, promoting blood circulation, and removing blood stasis. However, DM is treated with hypoglycemic Chinese and Western medicines. Although Xiaoke and DM overlap, they cannot be considered the same condition owing to the incongruency between them. A diagnosis of DM cannot replace a diagnosis of Xiaoke. The foundation and similarities and differences between Xiaoke and DM according to TCM are summarized in Fig. 3.

## Research advances in the mechanism of TCM treatment for DM

Modern research studies have shown that IR is the core mechanism of DM pathogenesis and it plays a vital role in the formation of DM. IR refers to a decrease in the effect of insulin on its target tissues, such as the brain, heart, liver, kidney, and skeletal muscle. The factors causing IR include: inflammation of the central nervous system, increased inflammation mediated by macrophages, increased concentrations of tumor necrosis factor (TNF-α), activation of the nuclear factor-κB (NF-κB) pathway, altered mitochondrial dynamics, increased levels of circulating fatty acids, and lipid accumulation in the muscles and liver [44–49]. Experimental studies have also shown that TCM reduces IR through multi-target action and has an apparent advantage in treating DM and chronic complications. Modern research findings provide crucial theoretical support and a clinical basis for the clinical application of TCM. Its mechanism of action can be summarized as either stimulating β cells to secrete insulin or increasing the number of insulin receptors, improving responsiveness and affinity, or inhibiting the secretion of insulin antagonists and promoting glucose degradation, which ultimately improves IR. TCM treatment for DM can effectively control blood glucose and reduce target organ damage by alleviating the effects of IR [50]. TCM in the treatment of DM includes many means, such as single Chinese herbal medicine, TCM compound, traditional chinese patent medicines and simple preparations, chinese medicine external treatment, acupuncture and moxibustion, and sports therapy (also including Taijiquan and Baduanjin). Chinese herbal medicines that are medicine and food homologies, such as Astragali Radix, Ginseng Radix et Rhizoma, Rehmanniae Radix, Coptidis Rhizoma, and their proven prescriptions, small compound prescriptions, active parts, and monomer components, have unique advantages for safety and effectiveness in the treatment of DM, which have been commended in recent years. Specific research examples are described below.

### Study on a single Chinese herbal medicine

The compositions of Chinese herbal medicines are complex. Chemical compositions and pharmacological activity of these medicines have been studied in the treatment of DM, combined with modern separation and analysis methods. Studies have mainly focused on Astragali Radix, Ginseng Radix et Rhizoma, Rehmanniae Radix, Pueraria Lobata Radix, Trichosanthis Radix, Dioscoreae Rhizoma, Coptidis Rhizoma, Mori Folium, Codonopsis Radix, and Ophiopogonis Radix. The details are as follows:

Astragali Radix is the dried root of *Astragalus memeranaceus* (Fisch.) Bge. Var. *mongholicus* (Bge.) Hsiao or *Astragalus membranaceus* (Fisch.) Bge., which has the following functions: lowers blood glucose, lowers lipids, has an anti-inflammatory effect, and improves islet function [51]. The main active ingredients are polysaccharides, saponins, flavonoids, and triterpenes. Astragalus polysaccharide can reduce glycosylated hemoglobin levels, blood glucose, and serum insulin. It can decrease the level of endoplasmic reticulum stress, improve the functional morphology of islets, and increase the number of islet β cells in DM rat models, to inhibit or delay apoptosis of the islet and protect its function. Astragaloside IV in Astragali Radix can reduce blood glucose, increase insulin sensitivity, and improve IR [52]. It also has a specific protective effect on the kidneys and can reduce DN fibrosis [53]. It can also increase the activity of fork-head box transcription factor 1 by inhibiting the phosphatidylinositol 3-kinase (PI3K)/protein kinase B (Akt) signaling pathway, which slows down the injury of renal tissue in patients with DM [54, 55]. Astragaloside IV can improve renal function and morphology of DM KK-Ay mice [56]. Astragali Radix can enhance immune functions [57] and can improve renal injury in DN by interfering with signal transduction and inflammatory reactions [58].

Ginseng Radix et Rhizoma is the dried root and rhizome of *Panax ginseng* C. A. Mey., which has pharmacological effects, such as enhancing learning and memory, strengthening the heart, anti-shock properties, enhancing immune function, and delaying aging. The main components are saponins, polysaccharides, polypeptides, and glycopeptides. Ginsenoside can improve the action of insulin; thus, lowering blood glucose. Ginseng glycopeptide (GGP) plays a hypoglycemic role by restoring the function of pancreatic β cells in DM rat models, reducing IR and glycolipid metabolism disorder, promoting liver glycogen synthesis, scavenging free radicals, and enhancing anti-lipid peroxidation [59, 60]. Ginsenoside Re can also selectively promote enzyme activity in the islet β cell plexus and acetylcholinesterase (AchE) activity in the hippocampus, thereby contributing to the prevention of DM [61]. Similarly, Ginsenoside Rh_2_ can stimulate nerve endings to release acetylcholine, activating islet cell M receptors and promoting insulin secretion. In alloxan DM mice treated with Ginsenoside Rb_3_, the degree of damage to the islet tissue was reduced, and cell morphology and structure were normal. In addition, Ginsenoside Rb_1_ also enhances the activity of islet β cells [62]. Ginseng Radix et Rhizoma improve hepatocyte glycogen synthesis, promote glucose utilization, and reduce gluconeogenesis, thereby lowering blood glucose [62]. The Ginseng aqueous extract can also regulate the mutual transformation of sugar and fat [63]. Further, the hypoglycemic movement of ginseng glycopeptide (GGP) is achieved by binding with a β-adrenoceptor to trigger the cyclic adenosine monophosphate (cAMP) signaling pathway and then promote aerobic glycolysis of mitochondrial glucose [64]. Ginseng polysaccharide GH1 can upregulate insulin release and increase plasma insulin levels in normal mice [65].

Rehmanniae Radix is the dried root tuber of *Rehmannia glutinosa* Libosch., which can lower blood glucose, protect the heart and liver, boost immunity, and provide an anti-tumor effect. Its main components include iridoids, phenylethanol glycosides, and saccharides [66, 67]. Catalpol, a component of raw Rehmanniae Radix, can effectively reduce blood glucose, protect islet cells, and increase insulin secretion in DM mice [68], as well as increase serum insulin levels, attenuate IR, alleviate inflammatory injury caused by DM and its metabolites [69], regulate lipid metabolism, and reduce the levels of cholesterol and triacylglycerol caused by a high-fat diet [70–72]. It can also effectively decrease fasting blood glucose (FBG) levels and 24 h urinary albumin excretion rate in T2DM mice models, ameliorate the pathological state of kidneys, reduce the damage to renal cells, protect the kidney [73, 74]. Both raw and processed Rehmanniae Radix can reduce blood glucose levels in T2DM mice models, prevent pathological damage and fibrosis in the pancreas, kidneys, and liver, and reduce inflammatory reactions. However, the mechanisms for regulating multi-organ energy metabolism and exerting anti-inflammatory effects to alleviate the symptoms in DM mice vary [75].

Pueraria Lobata Radix is the dried root of *Pueraria lobata* (Willd.) Ohwi, which effectively lowers blood levels of glucose, pressure, and lipids and enhances antioxidation. It comprises isoflavones, triterpenes, saponins, and polysaccharides [76]. Pueraria Lobata Radix plays a hypoglycemic role by protecting islet β cells, improving liver function, and activating the Akt pathway downstream of insulin receptors [77]. Pueraria can also reduce the levels of blood glucose and lipids in DM rats, inhibit IR, enhance the sensitivity of surrounding tissues to insulin, and promote insulin secretion by islet β cells. It can inhibit the expression of hepatic acetyl coenzyme A carboxylase (ACC) mRNA, alleviate IR in T2DM mice by regulating the hepatic ACC signaling pathway, and further promote glucose and lipid metabolism.

Trichosanthis Radix is the dried root of *Trichosanthes kirilowii* maxim. or *Trichosanthes rosthornii* Herms is an essential drug for treating DM and has pharmacological effects, such as anti-tumor, anti-virus, enhancing immunity, and lowering blood glucose levels. It mainly consists of trichosanthin (TCS), *trichosanthes kirilowii* lectin (TKL), and trichosanthin polysaccharide. TCS can prevent or treat DM by regulating the functional imbalance of Th1/Th2 cells. TKL improves the body’s antioxidant ability and regulates glucose and lipid metabolism [78].

Dioscoreae Rhizoma is the dried rhizome of *Dioscorea opposita* Thunb, whose effects include hypoglycemic, hypolipidemic, and antioxidant. The components are polysaccharides, Dioscoreae Rhizoma compounds, and amino and fatty acids [79]. Dioscoreae Rhizoma polysaccharides have the functions of reducing blood glucose levels, promoting insulin secretion, protecting islet function, and regulating lipid metabolism disorder in DM mice [80–82]. In DM mice models, it can reduce blood glucose levels induced by alloxan and exhibits a specific concentration of dependence. Moreover, Dioscoreae Rhizoma can effectively regulate cholesterol levels, triacylglycerol, and other related blood lipid indicators [83]; as well as reduce blood glucose levels, increase C-peptide values, and treat DM by increasing insulin secretion and improving the function of damaged islet β cells in DM rats [84]. It is also involved in the regulation of blood glucose, lipids, and antioxidation, and can improve insulin sensitivity [85].

Coptidis Rhizoma is the dried rhizome of *Coptis chinensis* Franch., C*optis deltoidea* C.Y. Cheng et Hsiao or *Coptis teeta* Wall., and functions in reducing blood glucose levels, regulating blood lipids, and anti-inflammatory effects. Alkaloids, coumarins, lignans, flavonoids, terpenoids, and polysaccharides are the main components. Berberine can reduce blood glucose, inhibit IR, enhance insulin sensitivity of surrounding tissues, promote insulin secretion, reduce lipids, cause anti-inflammation and antioxidation, reduce the production of advanced glycation end products, promote the secretion of glucagon-like peptide-1 (GLP-1), and increase the number of islets β cells [86, 87]. Furthermore, it can reduce liver IR through the miR-146b/SIRT1 pathway. It can also ameliorate hyperglycemia and hyperlipidemia in T2DM rats, reduce IR of the target tissue, and promote insulin secretion in islet β cells. Berberine can also prevent IR by increasing glucose consumption in a culture medium, increasing intracellular glycogen content, and upregulating leptin receptor mRNA expression levels of HepG2 cells.

Mori Folium is the dried leaves of *Morus alba* L., and functions in lowering blood glucose, regulating blood lipids, clearing away oxygen free radicals, and anti-virus activities. Its main components are flavonoids and flavone glycosides, polyphenols, and alkaloids. Mori Folium flavonoids can effectively reduce FBG and blood lipid levels. Insulin in T2DM rat models reduces the levels of serum free fatty acid (FFA) [88], inhibits apoptosis induced by FFA, improves energy homeostasis based on the expression of adenylate kinase 2 (AK2) protein and peroxidase proliferator-activated receptor γ coactivator 1α (PGC1α) protein, and has an anti-DM effect [60].

Codonopsis Radix is the dried root of *Codonopsis pilosula* (Franch.) Nannf., *Codonopsis pilosula* Nannf. var. *modesta* (Nannf.) L. T. Shen or C*odonopsis tangshen* Oliv., which regulates blood glucose, enhances immunity, has anti-hypoxia and anti-stress effects and delays aging. Its main components are saccharides, triterpenes, steroids, alkaloids, lignanoids, and flavonoids [89]. Codonopsis polysaccharide can scavenge free radicals in *vivo* and in *vitro* [90, 91] and reduce the damage caused by oxygen free radicals to islet β cells by enhancing antioxidant effects, protecting β cells, and attenuating IR, which reduces blood glucose levels in DM mice [92]. It can increase the cell proliferation ability of the diabetes vascular endothelial cell injury model, thereby inhibiting DM vascular endothelial cell injury [93], and its joint silencing information regulatory protein 4 (SIRT4) can inhibit oxidative stress and mitochondrial apoptosis, thus inhibiting DM vascular endothelial cell apoptosis in vitro.

Ophiopogonis Radix is the dried root tuber of *Ophiopogon japonicas* (L.f) Ker-Gawl., which can lower blood glucose levels, enhance immunity, delay skin aging, and provide anti-inflammation and anti-tumor effects. The main components are steroidal saponins, homoisoflavonoids, and polysaccharides. Ophiopogonis Radix polysaccharide can reduce blood glucose levels in mice with hyperglycemia induced by factors, such as glucose, adrenaline, and alloxan [94]; promote the transport and utilization of glucose by adipocytes, reduce FBG levels and inhibit IR in T2DM rats. It can also improve cardiac function; enhance the sensitivity of T2DM rats to exogenous insulin and the content of hepatic glycogen and skeletal muscle glycogen in blood; and significantly reduce the renal failure index, urinary albumin excretion rate, and pathological injury of pancreatic tissue in T2DM rats.

### Prescription studies

Owing to the complexity of the patient’s condition and the diversity of the pathogenesis, single-agent prescriptions have the disadvantage of limited efficacy and weak drug potency. TCM compound prescriptions under accurate syndrome differentiation are more suitable for clinical practice in complex pathogenesis and known diseases and syndromes. Classic compound prescriptions have promising therapeutic effects in DM, as described below.

Baihu decoction originates from the *Treatise on Cold Damage (Shang Han Lun)*, written by Zhang Zhongjing in the Eastern Han Dynasty. It comprises Gypsum Fibrosum, Anemarrhenae Rhizoma, Glycyrrhizae Radix et Rhizoma Praeparata cum Melle, and non-glutinous rice. It functions by reducing heat, generating body fluids, regulating blood glucose and lipid metabolism, reducing islet cell load, alleviating IR, and promoting insulin sensitivity. Baihu decoction can enhance insulin sensitivity in T2DM rats [95], lower blood glucose and lipid levels, improve glucose tolerance, reduce the level of serum inflammatory factors, and regulate liver lipid metabolism in T2DM rats by regulating insulin receptor substrate 1 (IRS-1)/PI3K/Akt signaling pathways [96, 97]. Renshen Baihu decoction can also prevent the progression of DN [98].

Banxia Xiexin decoction comes from the *Shang Han Lun*, written by Zhang Zhongjing, and is composed of Pinelliae Rhizoma, Zingiberis Rhizoma, Scutellariae Radix, Coptidis Rhizoma, Ginseng Radix et Rhizoma, Jujubae Fructus, and Glycyrrhizae Radix et Rhizoma. It regulates the following: the liver and spleen function, blood glucose and lipid metabolism, gut microbiota, and neurotransmitters [99]. Banxia Xiexin decoction can improve the proliferation and differentiation of islet β cells, prevent their functional failure, alleviate IR, regulate blood [100]. It can also enhance insulin sensitivity by regulating various cytokines such as TNF-α and interleukin-6 (IL-6), which are secreted by adipose tissue and cellular factors, thereby alleviating the inflammatory reaction and indirectly improving IR [101]. A high dose of Banxia Xiexin decoction can substantially reduce FBG levels and the IR index (IRI) [102]. There is no significant difference in efficacy between this decoction and metformin.

Erchen decoction is derived from the *Beneficial Formulas from the Taiping Imperial Pharmacy (Tai Ping Hui Min He Ji Jv Fang)*, written by Chen Shiwen in the Song Dynasty. It consists of Pinelliae Rhizoma, Pericarpium Citri Reticulatae, Poria, Glycyrrhizae Radix et Rhizoma Praeparata cum Melle, Zingiberis Rhizoma Recens, and Mume Fructus. It regulates Qi, resolves phlegm, reduces blood glucose, and produces an antioxidation effect [103, 104]. Erchen decoction can alleviate the symptoms of T2DM in rats with nonalcoholic fatty liver disease (NAFLD) by regulating the SIRT1/mitochondrial uncoupling protein 2 signaling pathway. Modified Erchen decoction can partially regulate blood glucose and lipid levels in DM rats with fatty liver disease and improve IR [105]. It can improve glucose tolerance, improve insulin sensitivity, and slow the progression of the pre-DM stage in patients. Further, this decoction can reduce body weight, inhibit IR, ameliorate blood glucose and lipids dysregulation, improve peripheral neuropathy, and promote the conduction velocity of sensory nerves and motor nerves in patients. It can also reduce high-risk cardiovascular factors, myocardial and renal function damage, and macro and microvascular diseases, as well as delay the incidence and development of DM complications. Moreover, it can also modify insulin signal transduction. Experimental studies (in *vivo* and in *vitro)* have reported that Erchen decoction and its modified prescriptions can attenuate IR by acting on the IRS-1/PI3K/Akt signaling pathways in hepatocytes [106].

The Yuquan pill is derived from *Ren-zhai’s Direct Guidance on Formulas (Ren Zhai Zhi Zhi Fang)*, written by Yang Shiying in the Song Dynasty. It comprises Pueraria Lobata Radix, Ophiopogonis Radix, Rehmanniae Radix, Schisandrae Chinensis fructus, Trichosanthis Radix, and Glycyrrhizae Radix et Rhizoma. It nourishes Yin, generates body fluid, relieves thirst and restlessness, invigorates Qi and regulates the stomach, lowers blood glucose, reduces the cellular level of pro-inflammatory factors, protects endothelial cells, and scavenges free radicals [107]. The ingredients in the Yuquan pill have hypoglycemic effects. Puerarin can improve blood glucose control in patients with T2DM and increase the body’s sensitivity to insulin. Ophiopogonis Radix polysaccharides can significantly inhibit blood glucose in insulin-dependent-alloxan mice models, indicating a protective effect against islet β-cell damage caused by alloxan. Glycyrrhizic acid and licorice flavonoids can inhibit α-glucosidase, delay the speed of intestinal absorption of glucose, and produce hypoglycemic effects. The hypoglycemic effect of Rehmannia polysaccharide in Radix Rehmanniae can have various mechanisms including glucose-dependent insulinotropic peptide and GLP-1 which promotes insulin secretion. Glucose-dependent insulinotropic peptide improves insulin sensitivity, while GLP-1 can inhibit glucagon secretion and promote insulin secretion. TKL in Trichosanthis Radix has anti-lipolysis and other insulin-like effects, and fructus Schisandra polysaccharides can regulate the expression of glucose transporter 4 in the cell membrane by upregulating the levels of Akt, PI3K, and IRS1, which then play an anti-IR role. The ingredients of the six herbs in the Yuquan pill can play a hypoglycemic role by improving insulin sensitivity [108, 109].

Danggui Liuhuang decoction comes from the *Secrets from the Orchid Chamber (Lan Shi Mi Cang)*, written by Li Dongyuan in the Jin and Yuan Dynasties. It comprises Angelicae sinensis Radix, Rehmanniae Radix, Rehmanniae Radix Praeparata, Scutellariae Radix, Coptidis Rhizoma, Phellodendri Chinensis Cortex, and Astragali Radix. It functions by clearing deficiency heat, nourishing Yin and purging fire, resisting liver fibrosis, and inhibiting islet cell apoptosis and immunosuppression. The substances contained in Danggui Liuhuang decoction, such as ferulic acid, Catalpol, Baicalin, Berberine, and Astragaloside IV, can promote glucose uptake in HepG2 cells, inhibit proliferation of T lymphocytes, promote the differentiation of regulatory T cells (Tregs) in *vivo*, inhibit the interactions between dendritic cells (DCS) and T lymphocytes, enhance the expression of α1-antitrypsin-1 (AAT-1), B-cell lymphoma gene-2 (Bcl-2) and cyclin D 1, inhibit the expression of Bcl-2 related X protein, and increase the expression of programmed death ligand-1 in DCS, which will delay the incidence and development of DM [110, 111].

Yu’nv decoction comes from *The Complete Works of [Zhang] Jing-Yue (Jing Yue Quan Shu)*, written by Zhang Jingyue in the Ming Dynasty, and is composed of Gypsum Fibrosum, Anemarrhenae Rhizoma, Rehmanniae Radix Praeparata, Ophiopogonis Radix, and Achyranthis Bidentatae Radix [112]. It functions by clearing stomach heat, nourishing kidney Yin, lowering blood glucose, anti-inflammation, and anti-ventricular remodeling. In the prescription, Anemarrhenae Rhizoma, Rehmanniae Radix Praeparata, and Radix Achyranthis Videntatae all improve T2DM symptoms and have antipyretic and anti-inflammatory effects. Anemarrhenae Rhizoma can lower blood glucose, reduce IR and blood lipid levels, and prevent atherosclerosis. Both diphenylpyrones mangiferin and neomangiferin in Anemarrhenae Rhizoma lower blood glucose levels and ameliorate T2DM symptoms. The flavonoid chemical constituents in Rehmanniae Radix have anti-inflammatory and antibacterial effects, and some glycosides can effectively lower blood glucose and improve blood lipid levels [66, 113]. Berberine in Radix Achyranthis Videntatae can improve glucose uptake and utilization, and reduce fat production, glycogen decomposition, and gluconeogenesis [114]. Triterpenoids and ecdysterone can prevent the increase in drug-induced blood glucose levels, reduce blood lipid levels, regulate immunity, and strengthen the heart [115–117].

Liuwei Dihuang’s decoction comes from the *Jing Yue Quan Shu,* written by Zhang Jingyue in the Ming Dynasty. Liuwei Dihuang decoction, composed of Rehmanniae Radix Praeparata, Corni Fructus, Dioscoreae Rhizoma, Moutan Cortex, Alismatis Rhizoma, and Poria, has the functions of nourishing the liver and kidney Yin, regulating immune function, and providing anti-tumor and anti-aging effects. It also prevents and treats abnormal glucose metabolism. Liuwei Dihuang decoction can reduce the blood glucose index, cholesterol, triacylglycerol (TG), and low-density lipoprotein content. It can also increase the range of high-density lipoprotein in T2DM rat models. Through upregulating the expression of phosphodiesterase 3B factor in adipose tissue, the Liuwei Dihuang decoction can promote Akt-mediated phosphorylation and activation, reduce the level of cAMP in cells, reduce the activity of protein kinase A, and ultimately reduce the hydrolysis of stored TG and the release of free fatty acids from adipocytes, to alleviate IR [118]. Its water-extract and alcohol-soluble parts can promote the recovery of insulin signaling in adipose tissue and intervene in IR by upregulating the expression of the crucial gene insulin receptor substrate 2, and PI3K and Akt in PI3K/Akt signaling pathways in the adipose tissue of T2DM rats, thereby improving T2DM [119]. In patients with T2DM, it can effectively reduce and maintain blood glucose levels, enhance the function of islet β cells, and further improve the quality of life [120].

The Jiaotai pill comes from the *Han’s Clear View of Medicine (Han Shi Yi Tong)*, written by Han Mao in the Ming Dynasty, which is composed of Coptidis Rhizoma and Cinnamomi Cortex. The Jiaotai pill is effective in the heart and kidneys and can treat insomnia caused by heart-kidney disharmony. Moreover, studies have found that the Jiaotai pill has therapeutic effects on T2DM [121], which can protect pancreatic islet β cells, promote insulin secretion [122], inhibit IR [123], regulate lipid metabolism [124], and promote anti-inflammatory effects [125]. Coptis chinensis attenuates IR by decreasing serum retinol-binding protein 4 levels and increasing glucose transporter 4 (GLUT4) levels [126]. Cinnamon extract increases glucose uptake in muscle and adipose tissues via GLUT4 production and transportation [127, 128], inhibits glycogen synthase kinase 3β by promoting glycogen synthesis in the liver [129], and lowers blood glucose levels by suppressing the gene expression of two gluconeogenic regulators in the liver [130].

The modified prescription of Fenugreek pills is used in tonifying kidneys, strengthening Yang, and in the detoxification method proposed by Professor Lu Fuer through adding and subtracting a combination of the Fenugreek and Jiaotai pills, composed of Trigonellae Semen, Coptidis Rhizoma, Cinnamomi Cortex and Achyranthis Bidentatae Radix. Of those, Trigonella foenum greacum L Saponin, Diosgenin, 4-Hydroxyisoleucine, Berberine and Cinnamaldehyde are studied more extensively and are reported to have an effect in elevating FBG levels and glucose and insulin tolerance. Concurrently, they can stimulate insulin secretion, increase insulin sensitivity, and alleviate the symptoms of diabetes and diabetic kidney disease. Most of these active ingredients produce a therapeutic effect through inhibiting oxidative stress and inflammation, and modifying the expression of related genes. While PI3K/AKT, ACC/CPT-1A and AMPK signaling pathways play important roles in these processes [131].

### Other therapies

Acupuncture and moxibustion therapy, guided by the theoretical concepts of TCM with the efficacy of treatment based on syndrome differentiation, have recently been widely used in treating DM. These therapies lower blood glucose, reducing the level of inflammatory factors, and attenuate IR. Electroacupuncture at the following acupoints: “Sanyinjiao,” “Housanli,” “Pishu,” and “Weiwanxiashu” can reduce the serum leptin levels in T2DM rats, inhibit leptin resistance, and alleviate IR [132]. Taijiquan is a recommended exercise in the Chinese Guidelines for the Prevention and Treatment of Type 2 diabetes, as it has been shown to regulate glucose and lipid metabolism, reduce inflammation levels, improve obesity and overweight conditions, and enhance cardiopulmonary function [133].

## Preparation of T2DM models for research on the mechanism

### Modeling methods of WM

In terms of DM animal models, WM has more established modeling strategies such as using chemicals to destroy islet β cells in animals to create a model of DM or using streptozotocin (STZ) or alloxan to induce hyperglycemia in animals. To create a rat model of obesity and DM, rats were injected with low-dose STZ to induce slight damage to islet β cells, resulting in abnormal glucose tolerance. As such, after feeding the animals a high calorie diet for 10 weeks, obese is induced confounded by hyperglycemia, hyperinsulinemia, and IR. Another type is the IR spontaneous diabetes animal model such as db/db and ob/ob mice, which have obesity and hyperinsulinemia similar to human T2DM. These animal models are often used to develop new oral drugs to treat DM and its complications [134].

In addition, DM models simulating damaged human pancreatic cells are created in *vitro*; such a model of DM was induced using a modified medium that contained a precise dose of glucose to act on mouse islet β cells or rat islet cell tumor cells [135, 136].

The use of chemical substances to specifically destroy islet β cells to induce DM is the most common modeling method because of its simplicity and high success rate. However, drugs that stimulate insulin secretion and insulin sensitizers have no hypoglycemic response in these models. There are many types of DM and the models achieve an experimental “drug-induced” DM simulation, and not actual clinical type of DM. Moreover, owing to the various etiologies, pathogeneses, and syndrome types in patients with T2DM/Xiaoke, there is currently no recognized animal model simulating TCM syndromes. Experimental studies often use spontaneous, induced, and transgenic animal models to simulate different syndromes of T2DM/Xiaoke.

### Modeling methods of TCM

After consulting the literature, it is evident that most animal model strategies of TCM are based on the modeling methods of WM and auxiliary means to simulate the pathogenic progression of the disease in humans. A TCM`s DM model of the syndrome of phlegm dampness, blood stasis, and heat was established using seven specific pathogen-free (SPF) 8-week-old Sprague–Dawley (SD) male rats using a 45% calorie-ratio high-fat diet combined with restricted activity space [137]. A TCM`s DM model of the syndrome of liver depression and spleen deficiency was established using SPF 6-week-old SD male rats fed fructose water and a high-fat diet. Simultaneously, the rats were bound in a supine position to restrict activity and prevent food and water intake for 3 h daily [138]. Feng Hui et al. studied SPF db/db mice in the WM IR-type spontaneous DM animal model at 8–14 weeks. This type of rodent met the syndrome characteristics of excess Yang and heat in the early stage of Xiaoke, combined with the syndrome of phlegm dampness and Qi deficiency; at 14–16 weeks, these rats displayed characteristics of a deficiency in spleen Qi and accumulation of phlegm and dampness. At 16–20 weeks, it exhibited characteristics of insufficiency of both the spleen and kidneys and deficiency of both Qi and Yin in the middle and late stages of Xiaoke disease, combined with the manifestations of syndromes of kidney Yang deficiency and phlegm dampness [139]. Lv Yiyan et al. used SPF ICR male mice that were each injected intraperitoneally with STZ solution for five consecutive days. The short-term model group was administered warm medicine on day 9 after the STZ injection. The long-term model group was administered the medicine for 35 consecutive days to establish the TCM`s DM model of internal heat Xiaoke syndrome [140].

A few modeling strategies exist for creating DM animal models in TCM. The reason may be that TCM emphasizes treatment based on syndrome differentiation. TCM requires clinical practice and research is performed on humans rather than animals. For TCM, the pathogenesis of human conditions cannot be mimicked in animals owing to inter-species differences that make it challenging to simulate syndrome differentiation and its complexity. However, considering the associated ethical issues, it is not ethical to conduct the research in humans. Therefore, modeling methods for TCM rely on multiple disciplines, such as zoology and veterinary medicine that also use TCM [141].

## New methods and technologies for research on TCM and anti-DM mechanisms

Recently, advancements have been made in the development of instrumental analyses and the introduction of systems biology platforms, multi-omics technologies (i.e., genomics), epigenetics, transcriptomics, proteomics, microbiomics, and metabolomics, as well as data analyses methods, such as network pharmacology and bioinformatics. Multi-omics technology can reflect the interactions between various substances in biological systems, which is consistent with the perspectives of TCM in many aspects. They all emphasize the characteristics of the whole, connections, constant changes, and key factors. Therefore, these new methods have been gradually applied to study the pathogenesis of DM.

### Genomics

Genomics is an interdisciplinary biological discipline for collective characterization, quantitative research, and comparative study of genomes in different organisms. It has since been applied to the study of DM. Through virtual screening of functional foods and analysis of their roles in regulating gene function, Afroz S et al. found that functional foods such as Rhizoma zingiberis recens, Rhizoma curcumae longae, and Momordica charantia can regulate DM complications induced by coronavirus infection through the regulation of the peroxisome proliferator-activated receptor γ (PPARG) and the TNF-α genes in DM [142]. Hu S et al. identified 1,366 DN-related differentially expressed genes from the GSE30528 dataset, including five target genes, i.e., KCNH2, NCOA1, KDR, NR3C2, and ADRB2, and then explored the action mechanisms of Radix salviae miltiorrhizae in the treatment of DN [143]. In a study by Zhao R et al., Pericarpium zanthoxyli showed a therapeutic effect on DM and comorbid conditions induced by oxidative stress when studying 280 intersecting genes and 105 signaling pathways and their related targets [144]. Dong Y et al. through the data analysis of genes, found that Radix astragali-Chinese angelica compound can treat DN by acting on vascular endothelial growth factor A, tumor antigen p53, IL-6, TNF-α, and mitogen-activated protein kinase (MAPK) 1 [145]. Genomics analysis has significantly contributed to related research on DM.

### Epigenetics

Epigenetics is a branch of genetics that studies heritable changes in gene expression that do not change the DNA sequence, including DNA methylation, histone modification, non-coding RNA, genomic imprinting, maternal effects, and gene silencing. It is a molecular bridge that connects genes and the environment. In recent years, studies have confirmed that the interactions between genetic and environmental factors play an essential role in DM pathogenesis. Kim A et al. found that the combined treatment of luteolin and fisetin can inhibit the production of cytokines in monocytes under a specific dose of glucose through epigenetic modifications, such as NF-κB activation and combination therapy may be a potential candidate for the treatment and prevention of DM and its complications [146]. Lee W et al. reported that gallic acid may potentially treat and prevent DM and comorbid conditions by inhibiting hyperglycemia-induced cytokine production in monocytes through epigenetic changes in NF-κB [147]. While a study by Jiang H et al. showed that baicalin inhibits the incidence of T2DM liver tumors by regulating methyltransferase 3/N6-methyl adenylate/hexokinase domain component 1 axis and downstream tyrosine-protein kinase p-JAK2/transcriptional activator-1/cysteine protease three pathways [148]. Epigenetics leads to persistent changes in gene expression; its application will provide a new perspective for the study in the incidence and development of DM, and it provides new focus areas and ideas for the clinical treatment of DM [149].

### Transcriptomics

Transcriptomics refers to a discipline that studies gene transcription and transcriptional regulation in cells. It has been used to further reveal the pathogenic molecular mechanisms of DM and its complications. Based on transcriptomic analysis, Folimu mori and Radix astragali can regulate the differentially expressed genes and proteins in the liver of STZ-induced DM mice, and the genes and enzymes involved in the chlorogenic acid biosynthesis pathway were identified [150]. Wild silkworms can be used to treat DM [151]. The curative effect of Huangqi Liuyi decoction in treating T2DM has been studied extensively [152]. Berberine in Gegen Qinlian decoction reportedly reduces blood glucose levels in DM rat model [153]. Mahongsu alleviated DN by activating podocyte protein phosphatase 2A [154]. Biomarkers used in the treatment of DM wounds by Shengji Huayu prescription have been identified [155]. Furthermore, 18 candidate drugs such as andrographolide, can be used to treat DR [156]. Therefore, transcriptomics can be used to study the active state of DM genes in different tissue cells and at different periods to corroborate DM treatment strategies.

### Proteomics

Proteomics is a science that takes the proteome as the research object and studies the protein composition and changes in cells, tissues, or organisms. The study of proteomics provides, not only a material basis for the principles of life activities, but also a theoretical basis and solutions for the elucidation and conquest of various disease mechanisms. As a new research method, it is widely used in the study of DM [157, 158]. The Zuogui pill medicated serum can prevent or ameliorate DM and comorbid diseases by regulating associated protein pathways in a culture medium [159]. Radix Trichosanthis and its protein components have hypoglycemic effects on DM mice by contributing in the insulin receptor pathway [160]. A study on the action mechanisms of Chinese herbal medicines in treating DM was previously performed [161]. A cell division control protein 42 homolog and the Ras homolog gene family member A were therapeutic targets of the Yiqi Yangyin Huatan Quyu prescription for T2DM [162]. Quercetin exhibits potentially therapeutic effects on T2DM by targeting the MAPK signaling pathway [163]. Ginseng can also treat DM and its complications by alleviating inflammation [164].

### Metabolomics

Metabolomics is a research method that quantitatively analyzes all metabolites in organisms and identifies a way to investigate the relative relationship between metabolites and physiological and pathological modifications. This method is an integral part of systems biology and has been applied in the study of DM. Song L et al. studied the therapeutic effects of extracts from cortex phellodendri and angelica dahurica on STZ-induced T2DM mice models based on gas chromatography-mass spectrometry (GC/MS) metabolomic profiling [165]. Zhu Y et al. conducted a fecal metabolomics study on DM mice treated with Ophiopogonis Radix polysaccharide MDG-1 [166]. Cui X et al. analyzed the mechanism of Scutellariae Radix and Coptidis Rhizoma to treat T2DM by studying plasma and urine [167]. Qin Z et al. revealed the hypoglycemic effects of Rehmanniae Radix, and Coptidis Rhizoma and their combination on high-fat diet-induced DM mice by performing metabolomics analyses on serum samples [168]. Wei H et al. found that urinary metabolomics combined with a personalized diagnosis under the guidance of TCM can determine subtypes of the pre-DM stage [169]. Through metabolomic analyses of intestinal microbiota and DM mice feces, Li CN et al. found that the combination of berberine and stachyose-induced glucose metabolism is better than berberine alone [170]. Yang L et al. used a non-targeted UPLC-LTQ-orbitrap metabolomics method to assess chemical characteristics of the extract and found that the Jinqi hypoglycemic preparation can alleviate T2DM [171]. Zhou S et al. used a UHPLC-HRMS-based metabolomic method for chemical identification and confirmed that different varieties of Momordica charantia can be used to treat DM [172]. Wang W et al. used a metabolomic method based on ultra-high performance liquid chromatography-quadrupole time-of-flight tandem mass spectrometry (UHPLC-QTOF/MS) to confirm the hypoglycemic effect of the Gegen Jiaotai pill on T2DM rats [173]. Meng X et al. used metabolomic examinations and reported that the Tiaowei Chengqi decoction can regulate body fat content and the content of medium- and long-chain fatty acids in the pancreas of obese mice, and they also found that the Tiaowei Chengqi decoction can treat DM by preventing obesity, inhibiting inflammation, and regulating metabolism [174]. Thus, metabolomics has unique advantages in the study of DM and its comorbid disorders.

### Microbiomics

Microbiomics is the systematic study of the sum of all microorganisms and their interactions in a specific environment and has been widely used in DM research. Berberine [175, 176], quercetin [177], and the Shenqi compound [178] can be used to treat metabolic diseases such as DM by regulating the gut microbiota and reducing intestinal glucose absorption. The effects of Baduanjin on the gut microbiota in patients with pre-DM stage was studied [179]. The impact of Erchen decoction on the gut microbiota and lipid metabolism disorder in Zucker-type DM obese rats was assessed [180]. Xiexin decoction can improve the symptoms in T2DM rats by regulating the gut microbiota [181]. Quercetin exerts protective effects on STZ-induced DPN rats through regulating intestinal microbiota and active oxygen levels [182]. Anemarrhenae Rhizoma can regulate intestinal microbiota and restore pancreatic function in DM rat models [183]. The anti-DM activity of Scutellariae Radix and Coptidis Rhizoma has been researched [184]. Tiaowei Chengqi decoction can regulate the gut microbiota and improve the symptoms of DN mice [185]. Microbiomics can be used to study the pathogenesis of DM by exploring modifications in intestinal microbiota as a strategy to develop treatments for DM.

### Network pharmacology

Using systematic network pharmacology and molecular docking methods, the pharmacological mechanism and material basis of berberine [176], Mori Folimu [186] and the Xiaoke pill [187] in the treatment of DM have been evaluated. The effective components and potential targets of the Liuwei Dihuang pill for the treatment of T2DM were predicted. The potential targets and molecular action mechanisms of Salviae Miltiorrhizae Radix et Rhizoma [188], compound Astragali Radix and Angelicae Sinensis Radix [145], and the Liuwei Dihuang pill [189] in the treatment of DN were studied. The mechanism of action of the Jiaotai pill in treating DM associated with depression was explored [190]. Combined with a validation experiment, the mechanism of Notoginseng Radix et Rhizoma [191] and taohong siwu decoction [192] in the treatment of DR was revealed, and the effects and components of Chinese herbal medicines Rhizoma Coptidis and Rhizoma pinelliae on diabetic gastroparesis were observed [193]. Through network pharmacology, a meta-analysis conducted to explore the potential molecular mechanisms of Rhizoma Polygonati in the treatment of DM and to provide a theoretical basis for the development and research of Chinese herbal medicines that are both medicines and food homologies for the treatment of DM [194]. The anti-DM effect of the Chinese herbal medicine flavonoid compound was systematically studied [195]. The safety and efficacy of TCM in treating T2DM patients were evaluated [196]. Potential TCM and associated active ingredient mechanisms in the treatment of DM were analyzed [197]. It was found that jinlida granules could improve blood glucose control and variability in newly diagnosed T2DM patients [198]. Therefore, network pharmacology overcomes the limitations of traditional research methods, plays an essential role in the analysis of DM data, and provides the possibility for in-depth research on the action mechanisms and treatment methods of DM and other diseases.

## Conclusion and prospects

With the advancements in living standards and changes in dietary patterns, the incidence rate of chronic metabolic diseases such as obesity, DM, hyperlipidemia, fatty liver disease, and osteoporosis is gradually increasing. Their harmfulness ranks second only to cardiovascular and cerebrovascular diseases and cancer, which adversely affect the quality of life. DM is one of the common chronic metabolic diseases. Currently, extensive research and consensus exist on the pharmacodynamic material basis and action mechanisms of TCM and WM in treating T1DM, T2DM, and GDM. However, only a few studies on DM are confounded by other metabolic diseases, such as T3DM and T3cDM. T3DM has similar symptoms to Alzheimer’s disease and DM. The deposition of β-amyloid peptide leads to a disorder of the insulin signal transduction pathway, resulting in the decline of insulin levels and IR, accompanied by apparent inflammatory mediator activation, oxidative stress, DNA damage, and mitochondrial dysfunction [199–201]. T3cDM, also known as pancreatic-derived diabetes mellitus, refers to DM secondary to pancreatic damage, including pancreatitis and pancreatic trauma, tumor, cystic fibrosis, as well as hemochromatosis [202]. Based on his long-term experience in the treatment of DM, Tong Xiaolin suggested that T3cDM is clinically characterized by a syndrome of stagnation of heat in the liver and stomach, and a syndrome of Qi stagnation and blood stasis, which can be treated with a modified dachaihu decoction. However, since then, there has been no further breakthrough in the treatment and research of T3cDM in TCM and modern medicine. Therefore, it presents a new area of study and potential research avenues in the pathogenesis of new types of DM and discovering effective therapeutic drugs or prescriptions and their effective mechanisms in TCM.

**Fig. 4:**
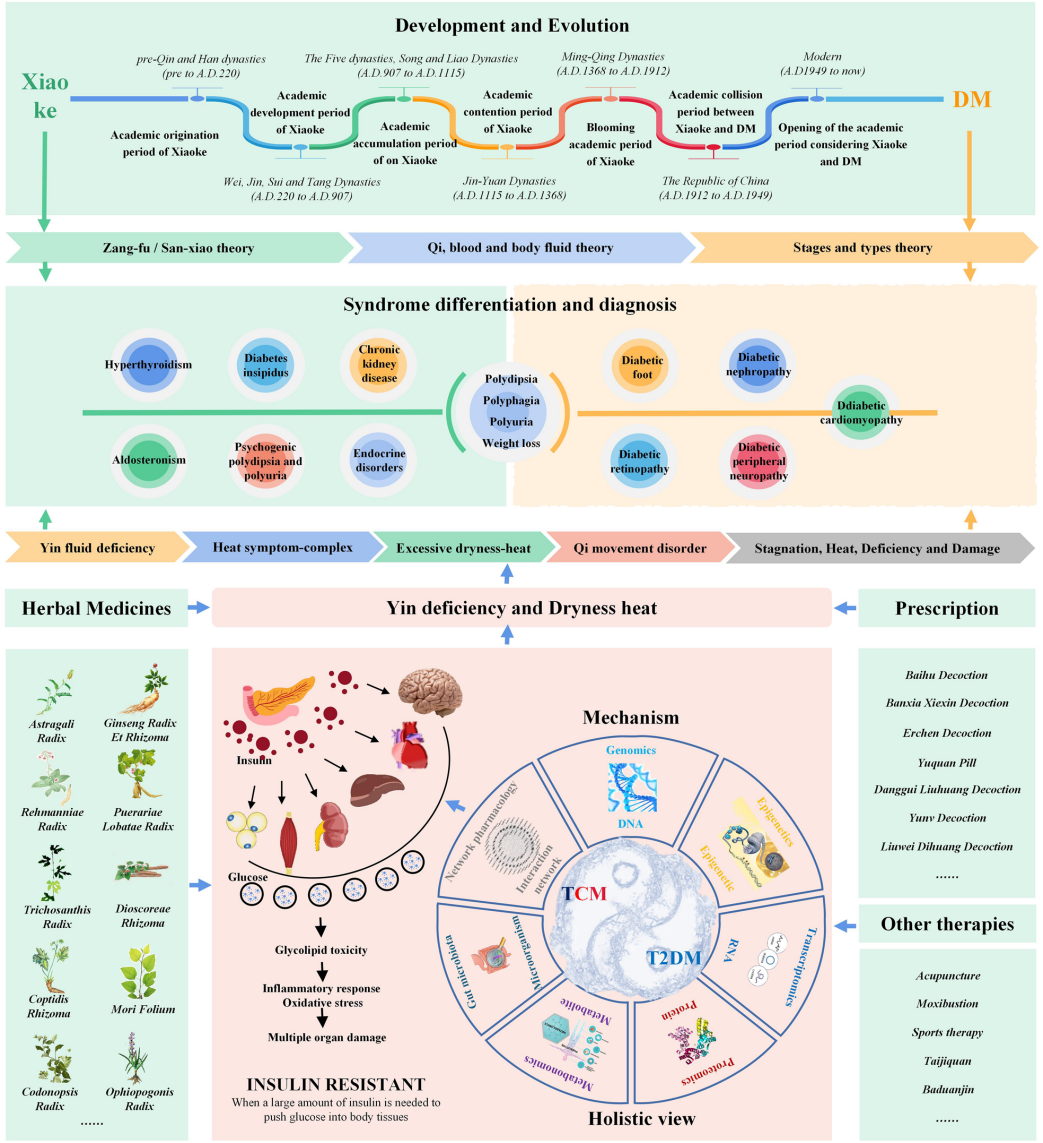
Summary of research progression in TCM for Xiaoke or DM treatment

The treatment principles and methods of DM in TCM are derived from Xiaoke. Guided by the principle of TCM, the treatment is based on syndrome differentiation, internal organs/Sanxiao (Qi, blood, and bodily fluids) and the disease stage. The treatment methods vary for each individual and are mainly focused on removing heat and nourishing Yin, replenishing Qi, generating bodily fluids, supplementing Yin, supporting Yang, promoting blood circulation, and removing blood stasis. TCM-based treatments are characterized by multi-channels, multi-targets, multi-links, significant curative effects, and fewer side effects. TCM has unique advantages in preventing the occurrence, development, and progression of DM (summarized in Fig. 4). Further studies should focus on therapeutic drugs and related action mechanisms in DM treatments, from screening single drugs, proven prescriptions, simple compound prescriptions, active and monomeric components with multiple hypoglycemic mechanisms, and improving insulin receptor sensitivity. Moreover, studies should establish animal models using different mechanisms, select corresponding control drugs, and explore new drugs with reasonable hypoglycemic effects that can prevent and treat DM complications. However, screening critical signaling pathways and core targets of TCM in the treatment of DM and clarifying its mechanisms of action are challenges hindering the advancement of TCM in the study of DM.

## Data Availability

Not applicable.

## Abbreviations

AAT-1: α1-Antitrypsin-1
ACC: Acetyl coenzyme A carboxylase
AchE: Acetylcholinesterase
AD: Alzheimer’s disease
AK2: Adenylate kinase 2
Akt: Protein kinase B
Bax: Bcl-2 related X protein
Bcl-2: B-cell lymphoma gene-2
cAMP: Cyclic adenosine monophosphate
DCS: Dendritic cells
DF: Diabetic foot
DM: Diabetes mellitus
DN: Diabetic nephropathy
DPN: Diabetic peripheral neuropathy
DR: Diabetic retinopathy
FBG: Blood glucose index
FFA: Free fatty acid
Fox O1: Fork-head box transcription factor 1
FPG: Fasting blood glucose
GC/MS: Gas chromatography-mass spectrometry
GDM: Gestational DM
GGP: Ginseng glycopeptide
GLP-1: Glucagon-like peptide-1
HDL-C: High density lipoprotein
IL-6: Interleukin-6
IL-6: Interleukin-6
INS-1: Islet cell tumor cells
IR: Insulin resistance
IRI: Insulin resistance index
IRS-1: Insulin receptor substrate 1
IRS2: Insulin receptor substrate 2
LADA: Type 1.5 diabetes
LDL-C: Low-density lipoprotein
MAPK: Mitogen-activated protein kinase
NAFLD: Nonalcoholic fatty liver disease
PDE3B: Phosphodiesterase 3B
PD-L1: Programmed death ligand-1
PDM: Pancreatic-derived diabetes mellitus
PGC1α: Peroxidase proliferator activated receptor γ coactivator 1α
PI3K: Phosphatidylinositol 3-kinase
PKA: Protein kinase A
PPARG: Peroxisome proliferator activated receptor γ
RhoA: Ras homolog gene family member A
SIRT1: Silencing information regulator
SIRT4: Silencing information regulatory protein 4
STZ: Streptozotocin
T1DM: Type 1 DM
T2DM: Type 2 DM
T3cDM: Type 3c DM
T3DM: Type 3 DM
TC: Cholesterol
TCM: Traditional Chinese medicine
TCS: Trichosanthin
TG: Triacylglycerol
TKL: Trichosanthes kirilowii lectin
TNF: Tumor necrosis factor
TP53: Tumor antigen p53
UCP2: Uncoupling protein 2
UHPLC-QTOF/MS: Ultra-high performance liquid chromatography-quadrupole time-of-flight tandem mass spectrometry
VEGFA: Vascular endothelial growth factor A
WM: Western medicine
